# SWEDEGENE—a Swedish nation-wide DNA sample collection for pharmacogenomic studies of serious adverse drug reactions

**DOI:** 10.1038/s41397-020-0148-3

**Published:** 2020-01-17

**Authors:** Pär Hallberg, Qun-Ying Yue, Erik Eliasson, Håkan Melhus, Joel Ås, Mia Wadelius

**Affiliations:** 1 Department of Medical Sciences, Clinical Pharmacology and Science for Life Laboratory, Uppsala University, Uppsala, Sweden; 2 WHO Uppsala Monitoring Center, Uppsala, Sweden; 3 Karolinska Institutet, Department of Laboratory Medicine, Clinical Pharmacology, Karolinska University Hospital, Stockholm, Sweden

**Keywords:** Risk factors, Genetics research

## Abstract

SWEDEGENE is a Swedish nation-wide sample collection established to facilitate studies of clinical and genetic risk factors for adverse drug reactions (ADRs). Most cases are recruited among patients reported to the ADR registry at the Swedish Medical Products Agency by health-care professionals. Clinical data are collected both from medical and laboratory records and through interviews using standardized questionnaires. Genome-wide scans and whole-genome sequencing are done, and association studies are conducted using mainly controls from the Swedish TwinGene biobank with data on diagnoses and prescribed drugs. SWEDEGENE was established in 2008 and currently contains DNA and information from about 2550 adults who have experienced specific ADRs, and from 580 drug exposed controls. Results from genome-wide association studies have now been published, and data from whole-genome sequencing are being analyzed. SWEDEGENE has the potential to offer a new means of developing individualized and safe drug therapy through patient pre-treatment screening.

## Introduction

Adverse drug reactions (ADRs) are a significant cause of morbidity and mortality, leading not only to individual treatment failures but also to substantially increased healthcare costs. ADRs have been estimated to cause or contribute to at least 5–7% of hospital admissions [1–4], and to about 3% of all fatalities [5]. About 10% of the Swedish health-care budget has been attributed to ADRs [6].

Virtually all drugs are unsafe in a subset of patients even when used according to the approved label. There is good reason to believe that a significant part of an individual’s risk of being intolerant to a drug is explained by genetic predisposition [7]. In some cases, dose-dependent ADRs are known to be caused by mutations in genes involved in the metabolism of the drug or in the drug target. An example is the dose-dependent bone-marrow suppression that develops in patients with defective detoxification of thiopurines related to genetic variants of the key enzyme thiopurine methyltransferase [8].

Other types of serious ADRs appear to be less dependent on dose. These ADRs—so called idiosyncratic or type B reactions—can affect various organ tissues, including e.g., the heart, liver, skin, kidney, and muscle or cause generalized hypersensitivity reactions [9]. In this category of ADRs, the cause is generally unknown and there are no obvious candidate genes. Microarray-based genotyping of multiple genetic variants as well as next-generation sequencing (NGS), where rarer sequence variants can be discovered, have made it possible to perform genome-wide association studies (GWAS) of these reactions, as well as other types of association analyses. For both of these methods, large numbers of patients are usually needed due to the statistical requirement of correction for multiple testing and for replication of findings.

In the relatively few large-scale genome-wide studies performed on serious ADRs so far, immune-related genetic variants involving the human leukocyte antigen (HLA) molecules in the major histocompatibility complex (MHC) on chromosome 6 have often been implicated as risk factors [10]. Such risk factors have been shown to be drug-specific and to vary between different ethnic populations. A well-known example is the association between HLA-B*57:01 and the abacavir-induced hypersensitivity syndrome [11]. Abacavir, a nucleoside reverse transcriptase inhibitor used in the treatment of human immunodeficiency virus (HIV), is associated with hypersensitivity reactions in 5–8% of patients [12]. Introduction of HLA-B*57:01 screening prior to abacavir therapy has reduced the incidence of this ADR from up to 8 to <1% [13].

In the current project, we are collecting ADRs on a large scale in Sweden. We aim to establish a large nation-wide DNA sample collection with clinical data to enable studies of both genetic and clinical risk factors of severe ADRs in order to improve the benefit/risk balance of drug treatment. The ultimate goal is to develop predictive tests and models that minimize the risk of severe ADRs, and thus reduce patient suffering and health-care costs. Such tests can also be used for diagnostic purposes to differentiate an ADR from spontaneous disease.

## Materials and methods

### Patient recruitment and data collection

SWEDEGENE (www.swedegene.se) was established in 2008 and is a Swedish nation-wide DNA sample collection with phenotype data on cases of ADRs. Most patients are identified and recruited through the Swedish national database of spontaneously reported ADRs run by the Medical Products Agency since 1965. Non-fatal cases reported from 1990 and onwards are extracted from the database, and each reporter is asked whether the patient can be approached about participation in SWEDEGENE. In addition, patients can be recruited directly from collaborating clinicians at health-care facilities. When a clinician at these collaborating centers identifies a suitable patient, the patient is either directly recruited in collaboration with SWEDEGENE, or a research nurse at SWEDEGENE will approach the patient and ask for participation. Another mode of recruitment is through advertising campaigns.

Population controls are obtained from the Swedish TwinGene biobank that has genome-wide data from over 10000 twins as well as whole-genome sequencing data from 1000 individuals born 1958 and before [14, 15]. Only one twin out of each pair is selected as a control. Through linkage with the Swedish Prescribed Drug Register and the National Patient Register kept by the Swedish National Board of Health and Welfare, diagnoses and drug prescriptions are matched between cases and controls. We also collect treated controls with full phenotype information directly from Swedish health-care facilities when necessary. The mode of recruitment for controls is identical to that for cases.

A study kit is provided to each consenting patient or treated control including a questionnaire holding information about demographics, medical history, environmental factors and information about drug treatment, as well as an informed consent form. A research assistant contacts the patient by telephone and the questionnaire is completed through a telephone interview. If needed, copies of the participant’s medical and laboratory records are obtained. In addition, blood samples are drawn at the patient’s nearest health-care facility, and sent to the central laboratory at Uppsala University Hospital, where they are stored for later use. If a patient is reluctant to draw blood, saliva sampling is undertaken instead.

Phenotype data concerning the drug suspected to have caused the ADR, the indication for which the drug was prescribed, concomitant drugs and diseases, a summary code for the ADR, demographic variables (sex and age), relevant laboratory data, a brief narrative, and all information acquired through the questionnaire is compiled in a study database by a research nurse. The same questionnaire is used for all cases except for certain ADR specific questions. For treated controls, the questionnaire contains demographic variables, all drug treatments and diseases. To ensure the security of participant data, the clinical data is stored in a local encrypted database. Access to the database is limited to specific computers, and access is locked behind passwords and two-factor authentication. To further limit the potential for data breach the user is only allowed to view essential data for the user-group, with access to other parts locked behind a permission system.

Any type of genetic data is pseudonymized and stored separate from clinical data. For smaller data volumes in the range 1–20 TB, data is archived on secured encrypted drives. Data that is currently being analyzed is stored on the UPPMAX Bianca Cluster at Uppsala University (www.upmax.se). Secure archiving of larger data volumes has not yet been needed. As the genetic analyses that are conducted are not approved as clinical tests in routine health-care, genomic results are not returned to participants.

### Inclusion and exclusion criteria

All patients included are at least 18 years of age and able to give informed consent. To be included in the study, the initial event should have occurred after the start of treatment and in some instances after withdrawal of the drug. Causality is assessed with the WHO standard algorithm [16]. Certain ADRs have specific inclusion and exclusion criteria and are adjudicated by clinical experts. Examples of such critera from published studies are given in Table 1.

**Table 1:** Examples of inclusion and exclusion criteria for cases of adverse drug reactions in published studies.

	Inclusion criteria	Exclusion criteria
Agranulocytosis [18, 19]	(1) An absolute neutrophil count of 0.5 × 10^9^/L (≤500/μL) or less during non-chemotherapy drug treatment or within 7 days of stopping treatment; (2) Complete recovery after cessation of the drug, with an absolute neutrophil count of more than 1.0 × 10^9^/L (>1000/μL) or a compatible bone marrow aspirate or biopsy finding.	(1) Anticancer chemotherapy within 1 month of onset of agranulocytosis; (2) Radiation therapy within the previous month; (3) Bone marrow transplantation at any time; (4) Ongoing infection with Epstein-Barr virus, hepatitis A virus, HIV, cytomegalovirus, or parvovirus B19; (5) Ongoing sepsis; (6) Ongoing miliary tuberculosis; (7) Current presence of chronic neutropenia (congenital cyclic or idiopathic); (8) Ongoing immunosuppressive therapy with cytotoxic drugs; (9) Current presence of malignant infiltration of bone marrow; (10) Hematological diseases (eg, myelodysplasia, aplastic anemia, pancytopenia, and other blood dyscrasias, such as hemoglobin ≤100 g/L or platelets ≤100 × 10^9^/L); (11) Current presence of systemic lupus erythematosus.
Angioedema due to ACE-inhibitors and ARBs [41]	(1) Symptoms in the head and neck region judged to be angioedema by a physician; (2) The initial event should occur during treatment with an ACE inhibitor or ARB; (3) It may recur after cessation of treatment.	1) Symptoms coinciding with urticaria; 2) Another likely cause such as severe facial trauma or infection; 3) Association with C1 inhibitor or complement deficiency (if this data is available); 4) Mutation in the C1 inhibitor (SERPING1) or factor XII (F12) gene (if this data is available).
Atypical femoral fractures due to bisphosphonates [22]	(1) The event should occur during or after bisphosphonate treatment; (2) The fracture must be located along the femoral diaphysis from just distal to the lesser trochanter to just proximal to the supracondylar flare; (3) At least four of the following features (a–e) must be present: (a) The fracture is associated with minimal or no trauma, as in a fall from a standing height or less; (b) The fracture line originates at the lateral cortex and is substantially transverse in its orientation, although it may become oblique as it progresses medially across the femur; (c) Complete fractures extend through both cortices and may be associated with a medial spike; incomplete fractures involve only the lateral cortex; (d) The fracture is noncomminuted or minimally comminuted; (e) Localized periosteal or endosteal thickening of the lateral cortex is present at the fracture site (“beaking” or “flaring”).	1) Fractures of the femoral neck, intertrochanteric fractures with spiral subtrochanteric extension, periprosthetic fractures, and pathological fractures associated with primary or metastatic bone tumors and miscellaneous bone diseases (eg, Paget’s disease, fibrous dysplasia).
Narcolepsy due to swine influenza A (H1N1) vaccination (Pandemrix) [21]	(1) Onset of symptoms after Pandemrix vaccination. **Narolepsy type 1:** (1) The patient has daily periods of irrepressible need to sleep or daytime lapses into sleep occurring for at least three months; (2) The presence of one or both of the following (a-b): (a) Cataplexy and a mean sleep latency of ≤8 min and two or more sleep onset REM periods (SOREMPs) on a multiple sleep latency test (MSLT) performed according to standard techniques. A SOREMP (within 15 minutes of sleep onset) on the preceding nocturnal polysomnogram may replace one of the SOREMPs on the MSLT; (b) Cerebrospinal fluid (CSF) hypocretin-1 concentration, measured by immunoreactivity, is either ≤110 pg/mL or <1/3 of mean values obtained in normal subjects with the same standardized assay. **Narcolepsy type 2:** (1) The patient has daily periods of irrepressible need to sleep or daytime lapses into sleep occurring for at least three months; (2) A mean sleep latency of ≤8 min and two or more SOREMPs are found on a MSLT performed according to standard techniques. A SOREMP (within 15 minutes of sleep onset) on the preceding nocturnal polysomnogram may replace one of the SOREMPs on the MSLT; (3) Cataplexy is absent; (4) Either CSF hypocretin-1 concentration has not been measured or CSF hypocretin-1 concentration measured by immunoreactivity is either >110 pg/mL or >1/3 of mean values obtained in normal subjects with the same standardized assay; (5) The hypersomnolence and/or MSLT findings are not better explained by other causes such as insufficient sleep, obstructive sleep apnea, delayed sleep phase disorder, or the effect of medication or substances or their withdrawal.	1) Onset of symptoms prior to Pandemrix vaccination.

*ACE* angiotensin-converting enzyme, *ARB* angiotensin II receptor type 1 blocker

### Genomic analysis

Power calculations for GWAS using a dominant genetic model show that 50 cases and 5000 controls give us 80% power to detect an odds ratio of 3–4 with a minor allele frequency of 40%, and 80% power to detect an odds ratio of 4–5 for variants with a minor allele frequency of 20%. This is based on the conventional genome-wide significance threshold of *p* < 5 × 10^−^^8^ [17].

## Results

To date, SWEDEGENE has DNA and curated clinical data from about 2550 individuals that have experienced specific ADRs. A list of collected ADRs as per July 2019 with at least 15 cases is presented in Table 2. We have also collected 580 drug-treated controls, and the largest group is methotrexate-treated rheumatoid arthritis patients showing no signs of liver toxicity, and individuals exposed to the swine influenza A (H1N1) vaccination Pandemrix without having developed signs of narcolepsy. However, for most ADRs comparisons are made with the 5000 populations controls with genome-wide data and 1000 with whole-genome sequencing data from TwinGene.

**Table 2:** Collected adverse drug reaction diagnoses with at least 15 cases presented in decreasing order of frequency as per July, 2019.

ADR diagnosis	Main suspected drug classes	*n*
Angioedema	ACE-inhibitors, angiotensin receptor blockers, immunosuppressants, NSAIDs, antibiotics	514
Liver toxicity	Antibiotics, immunosuppressants, NSAIDs, lipid lowering drugs, antiarrhythmics, antiepileptics, antidepressants, herbals, antithyroid drugs, alcohol abuse drugs, anticoagulants	461
Cytopenias (agranulocytosis, thrombocytopenia and anemia)	Antithyroid drugs, sulfasalazine, NSAIDs, antibiotics, immunosuppressants	270
Cough	ACE-inhibitors	152
Urticaria	Antibiotics, immunosuppressants, contrast media, NSAIDs, antiepileptics	152
Alanine aminotransferase (ALT) elevation in rheumatoid arthritis patients	Methotrexate	130
Anaphylaxis	Immunosuppressants, NSAIDs, antibiotics, iron	112
Central nervous system toxicity	Antibiotics, antimalarial drugs, antiviral drugs, interferon	94
Narcolepsy	H1N1 vaccine (Pandemrix)	83
Pancreatitis	Immunosuppressants, ACE-inhibitors, lipid lowering drugs, chemotherapy, antidiabetics	82
Renal toxicity	NSAIDs, antibiotics, immunosuppressants, antidiabetics, antiviral drugs, anticoagulants	70
Stevens-Johnson syndrome/toxic epidermal necrolysis	Antiepileptics, antibiotics, immunosuppressants, allopurinol	63
Osteonecrosis	Bisphosphonates	61
Atypical fracture	Bisphosphonates	58
Bleeding	Non-vitamin K oral anticoagulants (NOAC)	52
Myopathy/Rhabdomyolysis	Lipid lowering drugs	51
Other allergic reactions	Antibiotics, immunosuppressants, NSAIDs, antiepileptics, proton pump inhibitors	51
Phototoxicity	NSAIDs, antibiotics	50
Metabolic disorder/weight gain	Antidepressants, neuroleptics	46
Hyponatraemia	Antidepressants, antiepileptics	35
Hair loss	Immunosuppressants, anticoagulants, antiepileptics, lipid lowering drugs, ACE-inhibitors	28
Tendon rupture	Fluoroquinolones	25
Torsade de Pointes/QT-prolongation	Beta blockers, antidepressants, antiarrhythmics, antibiotics	22

In addition to the below numbers, a total of 580 drug-treated controls have been recruited

GWAS of agranulocytosis induced by antithyroid drugs or sulfasalazine, cough induced by angiotensin-converting enzyme (ACE) inhibitors, narcolepsy induced by Pandemrix and atypical femoral fractures induced by bisphosphonates have been published [18–22]. SWEDEGENE has also provided cases and controls in several other collaborative studies, such as GWAS and whole  exome sequencing  of statin induced myopathy [23, 24], drug induced liver toxicity [25–30], and hypersensitivity reactions to carbamazepine [31]. Additional GWAS and whole  genome exome  sequencing studies on ADR diagnoses collected by SWEDEGENE are currently underway. In addition, 1000 selected SWEDEGENE individuals have been whole genome sequenced and are  being compared with  1000 whole  genome sequenced individuals from TwinGene. This will give us the possibility to find novel genetic associations with ADRs and to map population frequencies of known pharmacogenomic targets in the Swedish population. As for planned analyses, enrichment tests [32], pathway based analysis, and genome-wide complex trait analysis [33] will be performed beyond GWAS.

As many pharmacogenomic targets are rare variants, novel associations in a limited sample population can be hard to detect. To increase the probability there are two options; increase the sample size or decrease the number of tested associations. As rare ADRs are, by definition, rare, our option is to decrease the number of tests. This will be done by selecting variants a priori based on predicted mutation effects using software and databases as Ensembl Variant Effect Predictor (VEP) [34], Eigen- [35] or Combined Annotation-Dependent Depletion (CADD) scores [36]. To further decrease the number of tests we will use enrichment tests such as burden and non-burden tests, to test for genetic burden on an exon or pathway basis [32].

### Future perspective

SWEDEGENE is an important resource for pharmacogenetic studies of ADRs. Due to our unique nation-wide collection, SWEDEGENE has the potential to discover novel genetic and clinical risk factors for rare and serious ADRs, with the ultimate goal to identify patients at risk and to improve the benefit/risk balance of drug treatment. Since pharmacogenetic variants in general have larger effect sizes than variants that increase the risk of complex diseases [37], the clinical benefit is estimated to be great. It is also easier to select an alternative drug than to modify risk factors for complex diseases. It has already been shown that hospitalizations and emergency department visits can be reduced by genotyping elderly polypharmacy patients [38], and that pre-prescription genotyping is cost-effective for certain ADRs [39]. Barriers for implementing genotype-based drug therapy will be overcome once patients have their genome readily available in the medical record [40]. This is estimated to happen within the near future through emerging Precision Medicine initiatives. SWEDEGENE is well placed to discover novel gene-ADR associations to be analyzed in these initiatives and invites collaborators for joint efforts.
